# Ultraviolet-B Radiation Stimulates Flavonoid Biosynthesis and Antioxidant Systems in Buckwheat Sprouts

**DOI:** 10.3390/foods13223650

**Published:** 2024-11-16

**Authors:** Xin Tian, Meixia Hu, Jia Yang, Yongqi Yin, Weiming Fang

**Affiliations:** 1 College of Food Science and Engerning, Yangzhou University, Yangzhou 210095, China; dx120230241@stu.yzu.edu.cn (X.T.); mz120222068@stu.yzu.edu.cn (M.H.); yqyin@yzu.edu.cn (Y.Y.); 2 Yangzhou Center for Food and Drug Control, Yangzhou 225009, China; jiajia82112001@163.com

**Keywords:** phenylpropane metabolism, functional food, gene expression, germination

## Abstract

Abiotic stress not only elevates the synthesis of secondary metabolites in plant sprouts but also boosts their antioxidant capacity. In this study, the mechanisms of flavonoid biosynthesis and antioxidant systems in buckwheat sprouts exposed to ultraviolet-B (UV-B) radiation were investigated. The findings revealed that UV-B treatment significantly increased flavonoid content in buckwheat sprouts, with 3-day-old sprouts exhibiting a flavonoid content 1.73 times greater than that of the control treatment. UV-B radiation significantly increased the activities of key enzymes involved in flavonoid biosynthesis (phenylalanine ammonia-lyase, 4-coumarate-CoA ligase, cinnamate 4-hydroxylase, and chalcone synthase) and the relative expression levels of the corresponding genes. Although UV-B radiation caused damage to the cell membranes of buckwheat sprouts, promoting increases in hydrogen peroxide and malondialdehyde content and inhibiting the growth of sprouts, importantly, UV-B radiation also significantly increased the activities of catalase, peroxidase, and superoxide dismutase as well as the relative expression levels of the corresponding genes, thus enhancing the antioxidant system of buckwheat sprouts. This enhancement was corroborated by a notable increase in ABTS, DPPH, and FRAP radical scavenging activities in 3-day-old sprouts subjected to UV-B radiation. Additionally, UV-B radiation significantly increased chlorophyll *a* and chlorophyll *b* contents in sprouts. These results suggest that UV-B radiation is advantageous for cultivating buckwheat sprouts with increased flavonoid content and enhanced antioxidant capacity.

## 1. Introduction

Buckwheat (*Fagopyrum esculentum* Moench.) is a gluten-free pseudocereal that belongs to the *Polygonaceae* family [1], and is grown extensively around the globe. Flavonoids, as secondary metabolites, have antibacterial, antioxidant, hypoglycemic, and antitumor effects [2,3,4]. Clinical research has demonstrated that the prolonged intake of buckwheat can markedly decrease blood lipid levels in mice models, enhance lipid metabolism, and diminish the incidence of fatty liver disease [5]. Extracts from buckwheat have the dual mechanism of inhibiting α-glucosidase activity and repairing pancreatic islet cell function, thereby improving glycemic indexes in type II diabetic rats [6]. These effects are attributed to the role of flavonoids in buckwheat. However, the current flavonoid content of buckwheat seeds only reached 630 μg/g FW, far from meeting the pursuit of health. Therefore, improving the flavonoid content of buckwheat has attracted great attention.

Germination promotes an increase in a variety of nutrients and secondary metabolites in young seedlings, including flavonoids and phenolic acids. It was found that the total phenols, total flavonoid content, and antioxidant capacity of buckwheat sprouts were higher than those of buckwheat seeds [7,8]. In addition, the production of various secondary metabolites is seen as a way for plants to adapt to environmental conditions [9]. It was shown that a light intensity of 315 μmol·m^−2^ s^−1^ notably increased flavonoid content, especially rutin, in buckwheat sprouts [10]. Treatment with low concentrations of sodium bicarbonate was found to notably enhance the flavonoid content of buckwheat sprouts and enhance the antioxidant capacity of buckwheat [11]. Sucrose treatment significantly increases flavonoid content and enhances antioxidant capacity in buckwheat sprouts [12,13]. In addition, changes in light source [14,15] and microwave treatment [16] are also found to significantly increase flavonoid content in buckwheat sprouts. Therefore, environmentally stimulated germination is considered to be an effective means of increasing secondary metabolites in plants.

Flavonoid biosynthesis begins with the phenylalanine metabolic pathway. This process involves crucial enzymes such as phenylalanine ammonia-lyase (PAL), cinnamic acid-4-hydroxylase (C4H), and 4-coumaroyl-coenzyme A ligase (4CL) [16]. The production and accumulation of flavonoids are closely linked to the expression levels of the genes encoding these enzymes. There is a significant positive linear correlation between PAL activity and flavonoid content in common buckwheat [17]. Research indicates that slight acid treatment can significantly increase black soybean C4H and 4CL activity and the related gene expression levels induce flavonoid enrichment [18]. sMethyl jasmonate and zinc sulfate treatments induce phenolic acid content in wheat sprouts by increasing PAL and C4H activities and upregulating *C4H* expression levels [19]. In addition, microwave irradiation stimulates the expression levels of PAL, chalcone isomerase (CHI), and flavanol synthase (FLS) and increases flavonoid concentrations in buckwheat sprouts [20]. Changes in the external environment are a potentially effective method to promote the flavonoid content in buckwheat sprouts.

UV-B, a common light radiation, not only eliminates foodborne pathogens or delays postharvest ripening processes after harvesting fruits [21], but also triggers significant changes in plant secondary metabolites, especially promoting the synthesis of flavonoids [22,23]. UV-B stress has been reported to induce and promote PAL activity and isoflavone synthesis in soybean sprouts [24,25]. A study found that UV-B radiation significantly increases phenolic content, especially the biosynthesis of quercetin flavonoids [26]. UV-B treatment significantly increases the accumulation of flavonoid monomers, especially epicatechin, in 40 buckwheat species [27]. The use of UV-B radiation with a wavelength band greater than 300 nanometers not only significantly increases the content of flavonoids in buckwheat sprouts, but also increases the content of anthocyanins [28]. Therefore, the enhancement of total flavonoid content in buckwheat sprouts by using UV-B irradiation has attracted increasing attention from researchers.

There is currently no information available regarding the accumulation of bioactive compounds and antioxidant properties in buckwheat sprouts exposed to UV-B radiation. In this study, the effects of UV-B radiation on flavonoid enrichment and antioxidant systems in buckwheat sprouts will be investigated in terms of physiological metabolism, enzyme activities, and expression levels of key genes to enrich the study of the effect of UV-B radiation on the physiology, biochemistry, and functional substances of buckwheat sprouts.

## 2. Materials and Methods

### 2.1. Cultivation of Buckwheat Sprouts

Buckwheat seeds were purchased from Taixing Institute of Agricultural Science, Taixing, China. The sterilized seeds were soaked in distilled water for 12 h at 25 °C, then evenly spread into boxes and placed in a germination chamber at 25 °C for germination. Different treatments were carried out: (1) CK: control, sprayed with distilled water; and (2) UV-B: a light bulb (30 μmol m^−2^ s^−1^) was used during germination according to a cycle of 8/16 h light/dark and sprayed with distilled water. For UV-B radiation, a narrowband UV-B lamp with a central wavelength at 313 nm (20 W, UVB-313, 290 nm–320 nm, Guangzhou Longpro Electric, Inc., Guangzhou, China) was used. These treatments were labeled CK and UV-B, and randomly sampled at specific germination times (0, 3, and 5 days) for analysis.

### 2.2. Physiological Metabolism

Thirty buckwheat sprouts were randomly selected, and the length and fresh weight of each sprout were determined using a straightedge (0.01 cm) and an analytical balance (0.001 g), respectively.

The malondialdehyde (MDA) and hydrogen peroxide (H_2_O_2_) contents were determined by the method of Yin et al. [29] with modifications. Buckwheat sprouts were ground with trichloroacetic acid (TCA), then centrifuged at 8000× *g*. Next, the supernatant was mixed with 0.67% thiobarbituric acid (V:V = 1:1) and heated in a boiling water bath for 30 min. After cooling, the absorbance of the solution was measured using a UV spectrophotometer (DR6000, Shanghai Ruishi Technology Co., Shanghai, China) to assess the MDA content.

Buckwheat sprouts were ground with PBS buffer and centrifuged. The resulting supernatant was combined with deionized water (V:V = 1:4), 1.0 mL of potassium iodide, and 1.0 mL of TCA, and then kept in the dark for 30 min. The absorbance was subsequently measured at 390 nm using a UV spectrophotometer (DR6000, Shanghai Ruishi Technology Co., Shanghai, China). A standard curve was created using H_2_O_2_ as a reference, which allowed for the determination of hydrogen peroxide concentrations in the samples based on this curve.

### 2.3. Total Phenolic and Total Flavonoid Contents

The total phenolic acid content was assessed following the method established by Kan et al. [30]. Specifically, sprouts were subjected to extraction using 50% (*v*/*v*) methanol. The extraction mixture was then centrifuged at a force of 10,000× *g* for a duration of 15 min to separate the soluble compounds. The resulting supernatant, measuring 1 mL, was carefully combined with 1.0 mL of 0.2 mM Folin–phenol reagent and 2.0 mL of 2% (*w*/*v*) sodium carbonate. This mixture was allowed to react for 2 h in darkness to prevent photo-degradation. The total phenolic content was subsequently quantified by measuring the absorbance at a wavelength of 765 nm with a UV spectrophotometer (DR6000, Shanghai Ruishi Technology Co., Shanghai, China). For calibration purposes, gallic acid served as the standard reference compound.

The flavonoid content was determined by Yin et al. [25], using rutin to create the calibration curve. The methanolic extract solution was combined with 2% aluminum chloride methanolic solution (V:V = 1:1). After allowing the mixture to incubate at room temperature for 15 min, the absorbance was measured at 430 nm using a UV spectrophotometer (DR6000, Shanghai Ruishi Technology Co., Shanghai, China). The total flavonoid content was reported in μg of rutin, based on comparisons with standard rutin that underwent the same treatment.

### 2.4. Antioxidant Capacity

Buckwheat sprouts were fully homogenized with 80% methanol and centrifuged; the supernatant was the solution to be measured. The determination of 1,1-Diphenyl-2-trinitrophenylhydrazine (DPPH) and 2,2′-azino-bis(3-ethylbenzothiazoline-6-sulfonic acid) (ABTS) clearance rate was conducted based on the work of Du et al. [31]. Specific details: 0.1 mL of supernatant was combined with 2.9 mL of DPPH, and incubated in the dark for 30 min, after which the absorbance at 517 nm was measured with a UV spectrophotometer (DR6000, Shanghai Ruishi Technology Co., Shanghai, China), and the result was expressed as DPPH clearance rate (%).

Similarly, 0.1 mL of supernatant was mixed with 2.9 mL of ABTS working solution, and incubated in the dark for 30 min, and then the absorbance value at 734 nm was determined with a UV spectrophotometer (DR6000, Shanghai Ruishi Technology Co., Shanghai, China). The result was expressed as ABTS clearance rate (%).

The FRAP (ferric ion-reducing antioxidant power) was determined with reference to Du et al. [32]. First, 0.25 mL of supernatant, 1.0 mL of phosphate buffer, and 1.0 mL of potassium ferricyanide were combined and incubated. Afterwards, 1.0 mL of 10% trichloroacetic acid (TCA) was introduced and thoroughly mixed. The absorbance was then measured at 700 nm using a UV spectrophotometer (DR6000, Shanghai Ruishi Technology Co., Shanghai, China), and the results were reported as the FRAP clearance rate (%).

### 2.5. Antioxidant Enzyme Activity

The activities of catalase (CAT), peroxidase (POD), and superoxide dismutase (SOD) were determined according to Yin et al. [25]. A single unit of activity for both CAT and POD was meticulously defined as a variation or change of 0.02 per minute when measured at optical densities of 240 nm and 470 nm (UV spectrophotometer, DR6000, Shanghai Ruishi Technology Co., Shanghai, China), respectively. A specific unit for measuring the activity of SOD was established, which was characterized by a variation of 0.02 per minute at optical densities of 560 nm (UV spectrophotometer, DR6000, Shanghai Ruishi Technology Co., Shanghai, China), correspondingly.

### 2.6. Flavones Synthetase Activity

PAL, C4H, and 4CL activities were determined according to the method of Ma et al. [33]. Buckwheat sprouts were subjected to a process of homogenization utilizing a Tris-HCl buffer solution, carefully adjusted to a pH of 8.9 and a concentration of 0.1 M. Following this procedure, the resulting homogenate underwent centrifugation at an acceleration of 12,000× *g* for 15 min, maintained at a temperature of 4 °C. This centrifugation step was conducted to isolate the supernatant, which contained the enzyme activities of interest. For this study, one unit of enzyme activity for PAL, C4H, and 4CL was defined as a measurable change of 0.01 per minute in optical density readings at 290 nm, 340 nm, and 333 nm with a UV spectrophotometer (DR6000, Shanghai Ruishi Technology Co., Shanghai, China), respectively.

CHI activity was carried out according to the method of Ji et al. [34]. The supernatant obtained from the prior extraction process was utilized as a source of crude enzyme. A precise volume of 0.75 mL of this crude enzyme solution was incorporated into a reaction mixture, which consisted of 2 mL of 0.05 M/L Tris-HCl buffer adjusted to a pH of 7.4, along with 7.5 mg/mL of bovine serum albumin, 50 mM/L of potassium cyanide, and 50 µL of a 1 mg/mL solution of hydroxylated chalcone. The enzymatic reaction was carried out under controlled conditions at a temperature of 30 °C for a duration of 30 min. To assess the activity of CHI, the variation in absorbance at a wavelength of 381 nm was monitored with a UV spectrophotometer (DR6000, Shanghai Ruishi Technology Co., Shanghai, China).

### 2.7. Chlorophyll Content

The method described by Zhang et al. [35] was used to determine the chlorophyll content in sprouts. The sprouts were homogenized with acetone and then centrifuged at 6000× *g* for 10 min. The supernatant was collected, and the absorbance was measured at 645 nm and 663 nm (UV spectrophotometer, DR6000, Shanghai Ruishi Technology Co., Shanghai, China).

### 2.8. RNA Extraction, Reverse Transcription, and Relative Gene Expression Levels

Total RNA was extracted from fresh buckwheat sprouts using a Plant RNA Extraction Kit (RC411, Vazyme, Nanjing, China). Total RNA was reverse-transcribed according to the PrimeScriptTM RT Master Mix Kit instructions (R423, Vazyme, China). The strip cDNA was quantified in triplicate using the SYBR Green method [36]. Table 1 lists the primers used in this study. The 2^−ΔΔCt^ comparative approach [37] was utilized to ascertain the relative expression levels of genes.

### 2.9. Statistical Analysis

All experiments were carried out in triplicate and the results are presented as mean ± standard deviation (SD). An unpaired *t*-test (CK and UV-B) or one-way analysis of variance (ANOVA) with Tukey’s test (1 d, 3 d, and 5 d) was performed with SPSS 21.0 software, considering *p*-values less than 0.05 to be statistically significant. Pearson correlation analysis was also conducted. The experimental data were processed using Excel 2010 software, and graphs were created using Origin 2022 software.

## 3. Results

### 3.1. The Growth Status, MDA Content, and H_2_O_2_ Content

**Figure 1:**
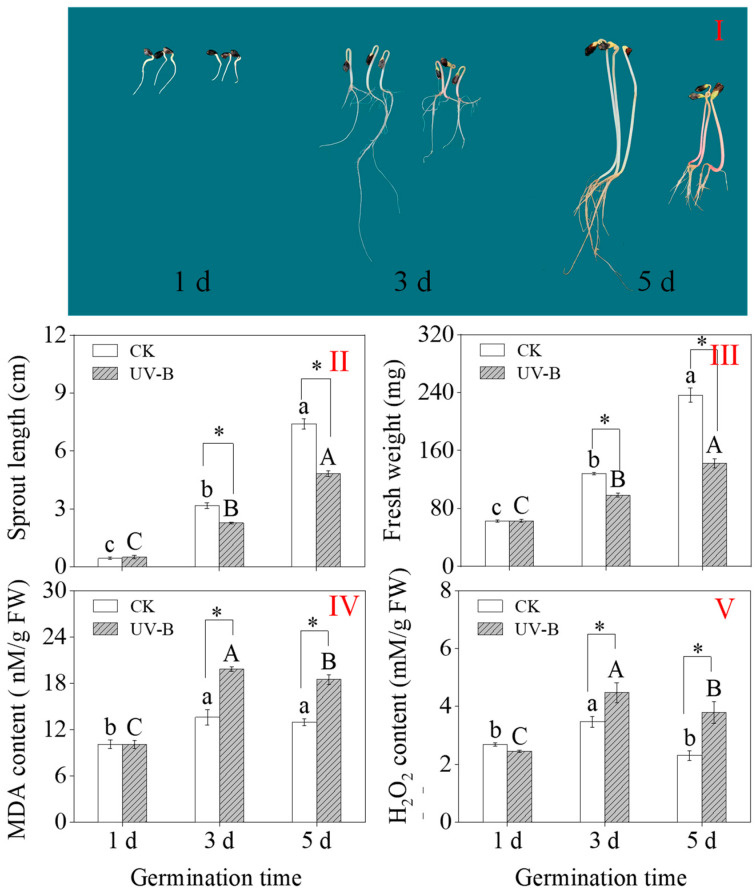
Effects of UV-B radiation on growth status (**I**), sprout length (**II**), fresh weight (**III**), MDA content (**IV**), and H_2_O_2_ content (**V**) of buckwheat sprouts. Different lowercase letters in the same index indicate significant differences between different germination times (1 d, 3 d, and 5 d) under CK treatment. Different capital letters indicate significant differences between different germination times (1 d, 3 d, and 5 d) under UV-B treatment. * denotes a significant difference between CK and UV-B radiation at the same germination time. CK: control treatment. UV-B: 8 h UV-B radiation. 1 d: one-day-old sprouts; 3 d: three-day-old sprouts; 5 d: five-day-old sprouts.

After three days, UV-B radiation inhibited sprout growth. Under UV-B treatment, the length of 3-day-old sprouts dropped by 28.1% and the length of 5-day-old sprouts dropped by 35.1% as compared to the CK (Figure 1II). Similarly, 3- and 5-day-old sprouts’ fresh weight was considerably decreased by UV-B radiation (Figure 1III).

The contents of MDA and H_2_O_2_ indicated the degree of stress to which the plants were exposed. As germination increased, the contents of MDA and H_2_O_2_ in the sprouts exhibited a tendency of first rising and then falling (Figure 1IV,V). Compared to the control, buckwheat sprouts that were 3 and 5 days old had significantly higher MDA and H_2_O_2_ contents after receiving UV-B treatment. Three-day-old buckwheat sprouts’ MDA and H_2_O_2_ contents increased by 45.58% and 28.81%, respectively, to their maximum values under UV-B exposure (19.82 nM/g and 4.47 nM/g, respectively). The findings demonstrated that UV-B treatment can considerably increase the MDA and H_2_O_2_ content of three-day-old buckwheat sprouts, which caused a stress effect on the sprouts, inhibiting the growth of sprouts and fresh weight.

### 3.2. Total Phenolics and Total Flavonoid Content

**Figure 2:**
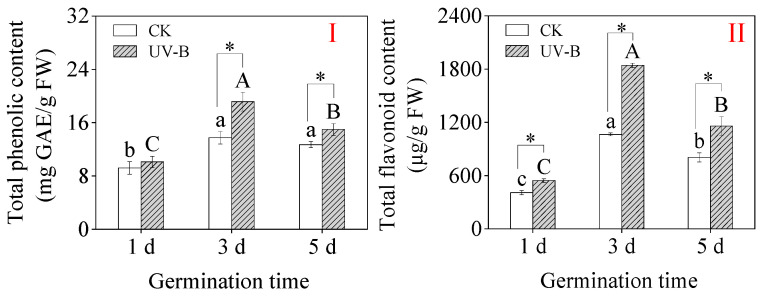
Effect of UV-B radiation on total phenolic content (**I**) and total flavonoid content (**II**) of buckwheat sprouts. Different lowercase letters in the same index indicate significant differences between different germination times (1 d, 3 d, and 5 d) under CK treatment. Different capital letters indicate significant differences between different germination times (1 d, 3 d, and 5 d) under UV-B treatment. * denotes a significant difference between CK and UV-B radiation at the same germination time. CK: control treatment. UV-B: 8 h UV-B radiation. 1 d: one-day-old sprouts; 3 d: three-day-old sprouts; 5 d: five-day-old sprouts.

Under CK treatment, total phenolics and total flavonoid contents were the highest in 3-day-old sprouts, which were 13.74 mg GAE/g and 1064.14 μg/g FW, respectively (Figure 2I,II). UV-B radiation dramatically raised the total flavonoid and total phenolic contents of 3- and 5-day-old sprouts as compared to the CK (Figure 2I). The 3-day-old buckwheat sprouts’ total phenolic and total flavonoid concentrations peaked at 19.14 mg GAE/g and 1842.33 μg/g FW, respectively, under UV-B treatment. The results showed that buckwheat sprouts’ total phenolic and total flavonoid contents were significantly raised by UV-B radiation.

### 3.3. Antioxidant Capacity

The clearance rates of DPPH, ABTS, and FRAP in 3-day-old sprouts were maximized under CK treatment (Table 2). UV-B radiation not only significantly increased the DPPH and ABTS clearance rates in sprouts (*p* < 0.05), but also significantly increased the FRAP clearance rate in 3-day-old sprouts (*p* > 0.05). Furthermore, it was observed that the DPPH, ABTS, and FRAP clearance rates were highest in 3-day-old sprouts treated with UV-B, reaching 75.71%, 67.42%, and 45.85%, respectively. The findings indicated that the antioxidant capacity was the strongest in 3-day-old sprouts and that UV-B radiation further increased the antioxidant capacity of 3-day-old sprouts.

### 3.4. Antioxidant Enzyme Activity and Relative Expression Level of the Corresponding Gene

**Figure 3:**
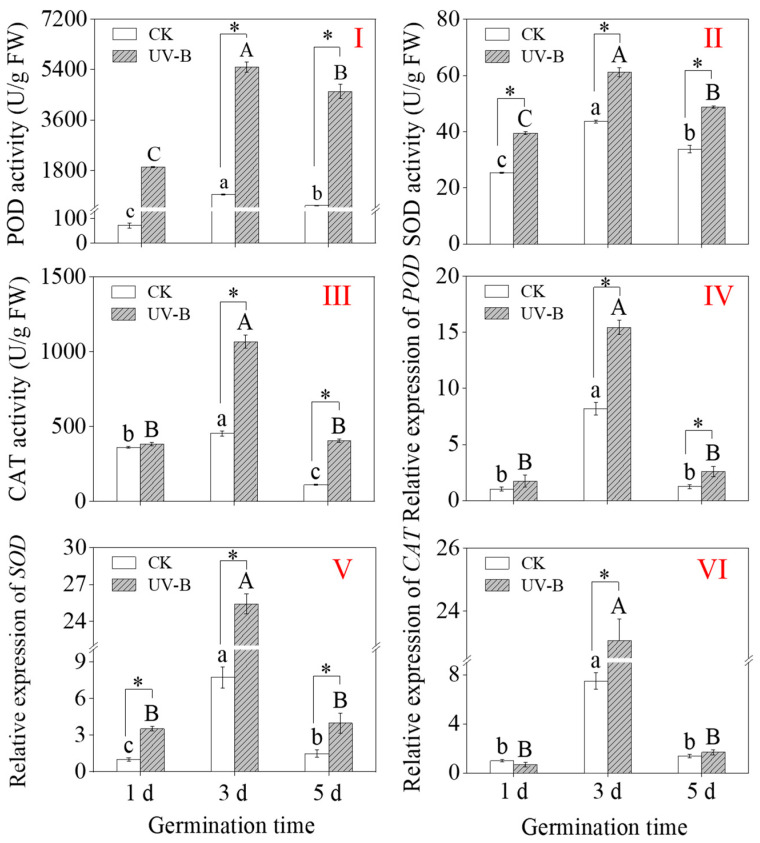
Effect of UV-B radiation on activity of POD (**I**), SOD (**II**), and CAT (**III**), and relative expression levels of *POD* (**IV**), *SOD* (**V**), and *CAT* (**VI**) in buckwheat sprouts. The relative expression level of individual genes in buckwheat sprouts germinated for 1 d under CK treatment was used as the control, and its value is 1.0. Different lowercase letters in the same index indicate significant differences between different germination times (1 d, 3 d, and 5 d) under CK treatment. Different capital letters indicate significant differences between different germination times (1 d, 3 d, and 5 d) under UV-B treatment. * denotes a significant difference between CK and UV-B radiation at the same germination time. CK: control treatment. UV-B: 8 h UV-B radiation. 1 d: one-day-old sprouts; 3 d: three-day-old sprouts; 5 d: five-day-old sprouts.

Compared with the CK, UV-B radiation markedly elevated POD, SOD, and CAT activities in 3-day-old and 5-day-old buckwheat sprouts (Figure 3I–III). Moreover, the maximum POD, SOD, and CAT activities in 3-day-old sprouts were 5489 U/g, 61.16 U/g, and 1067.65 U/g, respectively, under UV-B radiation. These values were 2.85-, 1.56-, and 2.79-times greater than those of the CK.

Under CK and UV-B treatments, 3-day-old buckwheat sprouts’ expression levels of *POD*, *SOD*, and *CAT* increased significantly (Figure 3IV–VI), and the highest multiplicative increase was observed. Compared with CK, UV-B radiation notably elevated the relative expression levels of *POD*, *SOD*, and *CAT* in 3-day-old buckwheat, which were 1.88-, 3.30-, and 5.7 times higher than CK, respectively (Figure 3IV–VI). The results showed that UV-B radiation significantly boosted the antioxidant systems in sprouts by enhancing the activities of antioxidant enzymes and the relative expression levels of associated genes.

### 3.5. Critical Enzyme Activities and Relative Expression Levels of Corresponding Genes for Flavone Synthesis

**Figure 4:**
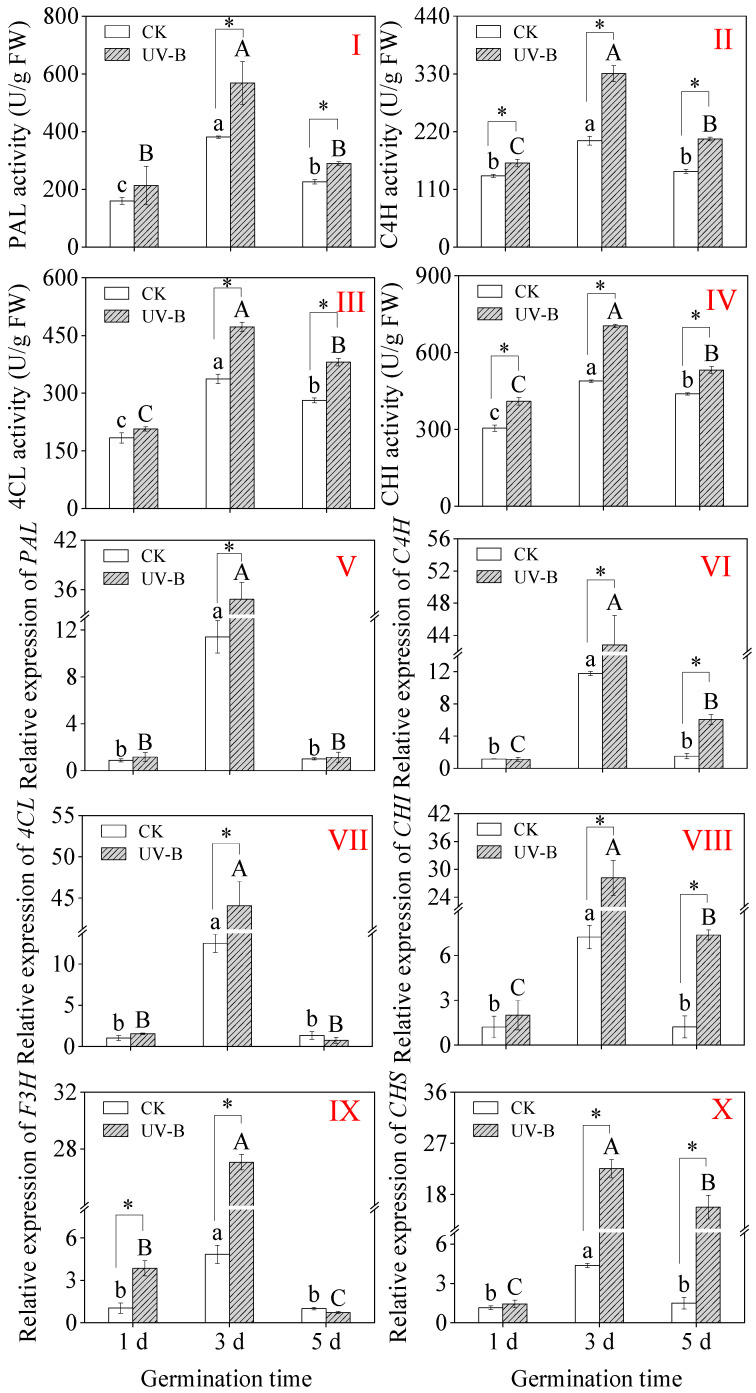
Effect of UV-B radiation on key enzyme activities ((**I**): PAL, (**II**): C4H, (**III**): 4CL, and (**IV**): CHI) and relative expression levels of genes ((**V**): *PAL*, (**VI**): *C4H*, (**VII**): *4CL*, (**VIII**): *CHI*, (**IX**): *F3H*, and (**X**): *CHS*) in buckwheat sprouts. The relative expression level of individual genes in buckwheat sprouts germinated for 1 d under CK treatment was used as the control, and its value is 1.0. Different lowercase letters in the same index indicate significant differences between different germination times (1 d, 3 d, and 5 d) under CK treatment. Different capital letters indicate significant differences between different germination times (1 d, 3 d, and 5 d) under UV-B treatment. * denotes a significant difference between CK and UV-B radiation at the same germination time. CK: control treatment. UV-B: 8 h UV-B radiation. 1 d: one-day-old sprouts; 3 d: three-day-old sprouts; 5 d: five-day-old sprouts.

As the germination process was prolonged, the sprouts’ PAL, C4H, 4CL, and CHI activity tended to rise and subsequently fall (Figure 4I–IV). UV-B radiation considerably increased the activities of C4H and CHI in one-day-old sprouts (Figure 4II,IV), with increases of 18.52% and 34.54% compared to the CK. The PAL, C4H, 4CL, and CHI activity were shown to be the highest under UV-B radiation in 3-day-old sprouts, at 568.56 U/g, 330.62 U/g, 472.23 U/g, and 704.13 U/g, respectively, which were 1.54-, 1.63-, 1.40-, and 1.44-fold greater than that of the CK (Figure 4I–IV).

The expression levels of metabolic enzyme-related genes were significantly higher in 3-day-old sprouts under CK treatment than in sprouts during the rest of the germination (Figure 4VI). While the expression levels of the other five genes did not change significantly (Figure 4V–VII,X), UV-B radiation significantly raised the expression level of F3H in 1-day-old sprouts compared to CK (Figure 4IX). Notably, the relative expression level of *4CL* was most affected by UV-B radiation, which was 3.65 times higher than that of CK treatment.

### 3.6. Chlorophyll Content and Expression Levels of UV-B-Related Genes

**Figure 5:**
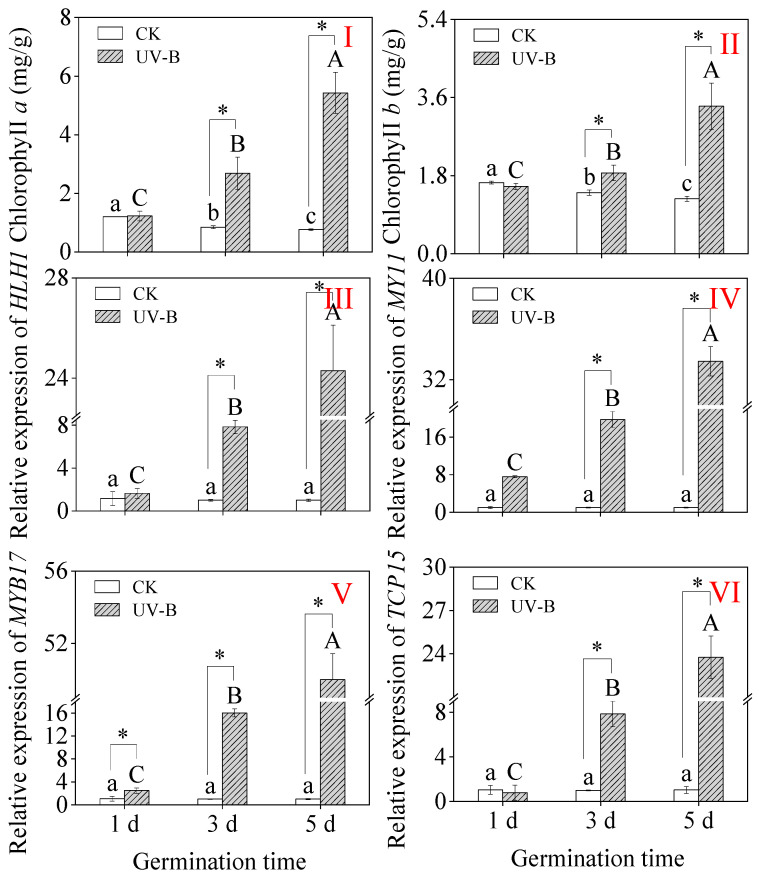
Effect of UV-B radiation on chlorophyll *a* content (**I**) and chlorophyll *b* (**II**) and the expression levels of UV-B-related genes (**III**–**VI**) in buckwheat sprouts. The relative expression level of genes of individual genes in buckwheat sprouts germinated for 1 d under CK treatment was used as the control, and its value is 1.0. Different lowercase letters in the same index indicate significant differences between different germination times (1 d, 3 d, and 5 d) under CK treatment. Different capital letters indicate significant differences between different germination times (1 d, 3 d, and 5 d) under UV-B treatment. * denotes a significant difference between CK and UV-B radiation at the same germination time. CK: control treatment. UV-B: 8 h UV-B radiation. 1 d: one-day-old sprouts; 3 d: three-day-old sprouts; 5 d: five-day-old sprouts.

Germination was able to promote a decrease in chlorophyll *a* and *b* contents under CK treatment as well as an increase in chlorophyll *a* and *b* contents under UV-B treatment (Figure 5I,II). In addition, UV-B treatment significantly increased the chlorophyll content in 3-day-old sprouts. UV-B treatment significantly increased the relative expression levels of genes (*HLH1*, *MY11*, *MYB17*, and *TCP15*) in sprouts at each growth stage. Moreover, with increasing germination time, the expression levels of these genes under UV-B treatment showed an increasing trend.

### 3.7. Correlation Between Indicators

**Figure 6:**
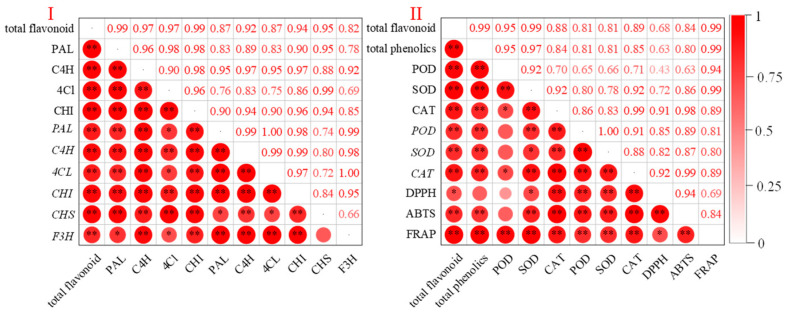
Correlation analysis of total flavonoid content with key enzyme activities and expression levels involved in flavonoid synthesis (**I**), and between antioxidant capacity and flavonoid anabolism-related indices (**II**) during buckwheat germination under UV-B treatment. Red hues indicate positive correlations. Asterisks (*) and (**) denote that the correlation coefficient is statistically significant at *p*-value thresholds of 0.05 and 0.01, respectively.

There was a significant positive correlation between flavonoid content and the activity of four synthesizing enzymes (Figure 6I, *p* < 0.01) and the expression levels of six genes (*p* < 0.01). Figure 6II depicts the correlation between the indices and antioxidant capacity under UV-B radiation. FRAP showed a significant correlation with all indices. ABTS clearance rate showed a significant positive correlation with total phenolic content (*p* < 0.01), total flavonoid content (*p* < 0.01), SOD and CAT activities (*p* < 0.01), and antioxidant enzyme gene expression levels (*p* < 0.01). DPPH clearance rate showed a significant correlation with the rest of the indices, except that it did not have any significant correlation with total phenolic content and POD activity (*p* > 0.05). DPPH was significantly correlated with all indicators except total phenolic content (*p* > 0.05) and POD activity (*p* > 0.05).

## 4. Discussion

In this study, it is found that UV-B radiation, as a good source of energy, significantly increased the total flavonoids in buckwheat sprouts and activated the antioxidant system. After germination, the nutrient content of buckwheat in the body will increase dramatically; research has shown that the free amino acid content and protein digestibility in germinated buckwheat are substantially increased, making it a good plant source of protein for the human body [38]. In addition, buckwheat contains a variety of bioactive compounds beneficial to human health, especially flavonoids. Flavonoids are known for their effectiveness in reducing blood lipids, enhancing immunity, and inhibiting the incidence of various cancers. As the medicinal properties of buckwheat flavonoids become increasingly recognized, enhancing the flavonoid content in buckwheat holds significant importance. Research has indicated that large doses of UV-B radiation can damage plants, but appropriate doses of UV-B irradiation are beneficial to organisms. UV-B at inappropriate doses enhances plants’ resistance to biotic and abiotic stresses [39,40] but also increases their secondary metabolites [41,42,43]. Studies have shown that optimal UV-B radiation levels can lead to an increase in secondary metabolite content in grapes [44,45], soybean sprouts [25], and other plants. Similarly, this study demonstrated that UV-B radiation can significantly increase the flavonoid and total phenol content in buckwheat sprouts, with flavonoid levels reaching up to 1842.32 μg/g FW in 3-day-old sprouts (Figure 2II). In addition, it was found that ABTS, DPPH, and FRAP clearance rates were significantly increased in sprouts under UV-B radiation (Table 2).

UV-B radiation negatively affects the permeability of plant cell membranes, thereby obstructing the typical growth and development processes in plants [46,47]. MDA is the primary byproduct of membrane lipid peroxidation and acts as a crucial marker for assessing membrane damage [48]. H_2_O_2_ exerts toxic effects on cells, and measuring its content can indicate the strength of a plant’s response to environmental stress, reflecting the extent of damage to plant tissues [48]. In this study, it was observed that buckwheat seedlings exposed to UV-B radiation during germination showed a significant increase in MDA and H_2_O_2_ content (Figure 1IV,V), leading to inhibited sprout growth. Throughout plant growth, specific defense mechanisms are formed within plants to counteract damage from abiotic stressors. These mechanisms include antioxidant enzyme systems and secondary metabolites to protect plant cell membranes from oxidative damage [9,49].

Flavonoids are effective substances produced by plants to resist UV-B radiation, capable of absorbing or filtering a portion of ultraviolet light to prevent plant damage [48]. It has been reported that UV-B radiation enhances the flavonoid content in Pelargonium graveolens, thereby increasing its antioxidant capacity [23]. Previous research has demonstrated that the ethanol extract of buckwheat sprouts exhibits significant reducing power and superoxide anion-scavenging activity, effectively inhibiting the production of peroxides within human liver cancer HepG2 cells and eliminating intracellular superoxide anions [50]. Treatments involving blue light [14], microwaves combined with l-phenylalanine [7], sucrose [13], and ultraviolet light [34] have resulted in buckwheat sprouts not only possessing the highest total flavonoid and total phenolic content but also exhibiting the greatest antioxidant activity and a strong relationship between the antioxidant potential of buckwheat sprouts and flavonoid concentration [51]. This study observed a significant increase in flavonoid content under UV-B treatment, along with a notable increase in ABTS, DPPH, and FRAP clearance rates (Table 2). In addition, a significant positive correlation was found between flavonoid content and DPPH clearance rate (*p* < 0.05), ABTS clearance rate (*p* < 0.01), and FRAP clearance rate (*p* < 0.01).

Studies have revealed that under UV-B treatment, soybean sprouts increase flavonoid content by upregulating PAL activity and the expression levels of *PAL* genes in the sprouts [25]. By significantly increasing the activities of PAL and C4H, as well as the gene expression level, γ-aminobutyric acid induces the flavonoid content in soybean sprouts under UV-B treatment [52]. CHI, as a key enzyme in the pathway of flavonoid biosynthesis, influences the biosynthesis of flavonoids through its gene expression level. Research on soybean callus tissues revealed that UV-B radiation not only upregulates the expression level of the *PAL* gene but also increases the expression levels of the *CHI* gene, enhancing their activity in soybean hypocotyls and cotyledon callus tissues. Identification through high-performance liquid chromatography showed a significant increase in the content of dihydrodaidzein in soybean callus tissues, thereby promoting the content of flavonoids [53]. The expression level of *CHI* is closely associated with the accumulation of anthocyanins in *Allium fistulosum*. It has been observed that the expression levels of *CHI* and *CHS* are highly synchronized, and further confirmed through yeast two-hybrid analysis and bioluminescence complementation imaging that *CHI* interacts physically with *CHS*. Additionally, a biosynthetic system involving *CHS* in the production of naringenin was constructed, indicating that the synergistic action of *CHS* and *CHI* enhances naringenin production [54]. Microwave radiation treatment significantly upregulates the transcription of various flavonoid biosynthetic enzyme genes in bitter buckwheat sprouts, thereby promoting flavonoid accumulation [20]. The above research findings indicate that the accumulation of flavonoids is promoted through synergistic interactions between synthases or synthase genes. In this study, it was observed that under UV-B radiation, the biosynthesis of flavonoids in buckwheat seedlings exhibited a similar trend with the metabolic enzymes PAL, C4H, 4CL, and CHI, showing a significant positive correlation. Moreover, significant positive correlations were found between changes in the expression levels of each gene and the activity of related enzymes.

Exogenous melatonin enhances the antioxidant capacity of soybean seedlings under ethylene treatment by increasing the activities of POD, SOD, and CAT, as well as upregulating the expression levels of *SOD* and *CAT* genes [36]. The antioxidant enzymes in plants primarily include SOD, CAT, and POD. These enzymes work synergistically to rapidly eliminate free radicals generated during plant metabolism. SOD is widely present in both animals and plants, serving as an enzyme that eliminates superoxide anion radicals. Research on mung bean seeds treated with UV-B radiation revealed a significant induction of antioxidant enzyme activity under UV-B treatment [55]. Furthermore, studies on cucumber [56] and grape [57] have shown that unfavorable conditions can modulate the activity of antioxidant enzymes and the expression levels of related genes, enabling resistance against oxidative damage. Although the expression levels of antioxidant enzymes and corresponding genes were not synchronized, this may be due to differences in plant species and varieties, site, growth stage, and mode and intensity of stress. In this study, it was observed that UV-B radiation significantly increased the activities of antioxidant enzymes and the expression levels of genes in buckwheat sprouts. Additionally, the correlation analysis of antioxidant capacity during buckwheat sprout growth under UV-B radiation revealed a significant positive correlation between the activities of SOD and CAT, the expression levels of POD, SOD, and CAT genes, and DPPH, ABTS, and FRAP. These findings suggest that UV-B treatment enhances the antioxidant capacity of sprouts by upregulating the activities of antioxidant enzymes and gene expression levels.

## 5. Conclusions

In summary, UV-B radiation effectively enhances the total flavonoid content and antioxidant capacity of buckwheat sprouts. This study found that UV-B radiation boosts flavonoid biosynthesis by significantly elevating the activities of enzymes such as PAL, C4H, 4CL, and CHI, while also upregulating the expression of their associated genes. In addition, UV-B radiation improves the antioxidant system by increasing the activity of antioxidant enzymes and their gene expression, thereby enhancing the resistance of buckwheat sprouts to UV-B exposure. This method is important for increasing flavonoid content and antioxidant capacity in buckwheat food.

## Figures and Tables

**Table 1:** Sequence-specific primers used in the present study.

Gene	Forward Primer (5′–3′)	Reverse Primer (5′–3′)
*Actin*	TCGTGAGAAGATGACCCAGA	CCGAGTCCAGCACAATACCT
*PAL*	TCTCCAGAAGCCGAAACAAG	AGCCTTGTTCCTGGATACAT
*C4H*	AACACACTACTCTCAGTTGC	ATTGGGTGATCGAGACTCTT
*4CL*	CTCTTTCACGTCCACGGTTT	GATGATTTGGTGGATGGTGG
*CHS*	CGTCAAGCGTTTCATGATGT	CAAGGCTTGTGTTGACATGG
*CHI*	ACTTTGAGGAATCCGCTGTGAC	AGGGCTTCAACATGGTGATCTGTA
*F3H*	CAAGGCTTGTGTTGACATGG	GACAGTGATCCAGGTCTTGC
*CAT*	GAGTTTGGTTCCCTTGCTT	TTCATACACTTCACTGGCGT
*SOD*	ATGGTGCTCCTGACGATG	CCACTGCCCTTCCAATAAT
*POD*	GTTCTGGTTGGGCTTGG	TTGTCCTCGTCTGTTGGTC
*TCP15*	GATAGGCTTGGCTATGATAGGCC	CAAACACAAATCTCGATGTGGGT
*HLH1*	TGTACGGATGTGGTTGAAACAT	TGTTCGTGAGCTGATGAACAAAGT
*MYB11*	GGTGGTCAATCAGCTCAGCCCA	TCGGTCCTACCTGGGAAGGCGAGC
*MYB17*	AGGAGGCAAGGTGGTTGTGGA	CATACCCATTAGCAAAGGAGAAA

**Table 2:** Effect of UV-B radiation on antioxidant capacity of buckwheat sprouts.

Categories		DPPH Clearance Rate %	ABTS Clearance Rate %	FRAP Clearance Rate %
1 d	CK	34.25 ± 3.08 c	40.72 ± 1.23 b	13.03 ± 0.93 c
	UV-B	46.94 ± 2.77 B *	45.29 ± 0.47 B *	13.59 ± 1.23 C
3 d	CK	59.56 ± 0.57 a	55.61 ± 1.01 a	34.91 ± 3.70 a
	UV-B	75.72 ±0.82 A *	67.42 ± 2.61 A *	45.85 ± 2.43 A *
5 d	CK	44.56 ± 0.49 b	29.59 ± 3.40 c	24.71 ± 0.90 b
	UV-B	49.33 ± 0.46 B *	41.53 ± 1.55 C *	28.54 ± 2.61 B

Note: Different lowercase letters in the same index indicate significant differences between different germination times (1 d, 3 d, and 5 d) under CK treatment. Different capital letters indicate significant differences between different germination times (1 d, 3 d, and 5 d) under UV-B treatment. * denotes a significant difference between CK and UV-B radiation at the same germination time. CK: control treatment. UV-B: 8 h UV-B radiation. 1 d: one-day-old sprouts; 3 d: three-day-old sprouts; 5 d: five-day-old sprouts.

## Data Availability

The original contributions presented in this study are included in the article. Further inquiries can be directed to the corresponding author.
